# LncRNA HCG11 Facilitates Nasopharyngeal Carcinoma Progression Through Regulating miRNA-490-3p/MAP3K9 Axis

**DOI:** 10.3389/fonc.2022.872033

**Published:** 2022-04-07

**Authors:** Jian Zheng, Zhuochen Zhao, Huijun Ren, Yongfeng Wang, Xianchun Meng, Wenjing Zhang, Cai Zhang, Liang Ming, Xiubo Lu

**Affiliations:** ^1^ Department of Thyroid Surgery, The First Affiliated Hospital of Zhengzhou University, Zhengzhou, China; ^2^ Department of Clinical Laboratory, The First Affiliated Hospital of Zhengzhou University, Zhengzhou, China; ^3^ Key Clinical Laboratory of Henan Province, Zhengzhou, China

**Keywords:** HCG11, miR-490-3p, MAP3K9, nasopharyngeal carcinoma, nasopharyngeal carcinoma progression

## Abstract

**Purpose:**

Long noncoding RNAs (LncRNAs) play complex but important roles in the progression of various tumors. This study aimed to elucidate the functional mechanisms of the HLA complex group 11 (HCG11) in nasopharyngeal carcinoma (NPC).

**Patients and Methods:**

HCG11 levels in NPC specimens were determined by fluorescence *in situ* hybridization (FISH) and qPCR. Proliferation, apoptosis, and metastasis of NPC cells were determined using CCK8, colony formation, annexin V-PI, and transwell assays. A murine tumor xenograft model was used to investigate the regulatory function of HCG11 in NPC *in vivo*, and immunohistochemical staining was used to determine the Ki-67 level in tumors. The target relationships between HCG11, microRNA miR-490-3p, and MAPK kinase kinase 9 (MAP3K9) were detected using bioinformatics, qPCR, western blotting, and luciferase reporter assays.

**Results:**

HCG11 was highly expressed in NPC tissues and was positively associated with tumor stage, lymphatic metastasis, and poor prognosis. Functionally, HCG11 knockdown inhibited proliferation and migration and induced apoptosis of NPC cells. Mechanistically, miR-490-3p is a direct target of HCG11, oncogenic functions of HCG11 in NPC cell proliferation and migration can be partially reversed by the miR-490-3p inhibitor. HCG11 significantly increased mitogen-activated protein kinase MAPK kinase 9 (MAP3K9) levels by inhibiting miR-490-3p.

**Conclusion:**

HCG11 facilitates NPC progression *via* MAP3K9 signaling by sponging miRNA-490-3p, which may contribute to new prognostic markers and promising therapeutic targets.

## Introduction

Nasopharyngeal carcinoma (NPC) is a head and neck tumor originating from the nasopharynx and is widespread in Asia (1). Patients with NPC are usually treated by surgical removal, radiation therapy, and chemotherapy, depending on the size and extent of the tumors (2, 3). Although the clinical treatment and management of NPC vary at present, the prognostic condition of NPC patients is still poor because of local invasion and metastasis (1). Therefore, it is necessary to investigate the underlying mechanisms of NPC development and metastasis (4).

Cancer is a multifaceted disease involving various epigenetic alterations, chromosomal translocations, genetic mutations, and amplification (5, 6), particularly long non-coding RNAs (lncRNAs) and microRNAs (miRNAs) (7). Accumulating studies have shown that lncRNAs and miRNAs are particularly expressed in different types and pathophysiological stages of tumors, making them promising molecular diagnostic markers for a variety of cancers. As emerging transcripts, lncRNAs are over 200 nucleotides in length and are rarely translated into proteins (8). At present, lncRNAs have been found to regulate chromatin dynamics, gene expression, tumor progression (proliferation, apoptosis, metastasis), cell metabolism and drug-resistance (7, 9). lncRNAs can modulate oncogenic and tumor-suppressing pathways by acting as competing endogenous RNAs (ceRNA) to competitively bind to miRNAs (7, 9, 10). For example, the lncRNA ARHGAP18 promotes tumor metastasis in HCC *via* miR-153-5p (11). HNF1A-AS1 is induced by MYC to facilitate glioma progression through miR-32-5p/SOX4 (12). H19 lncRNA is involved in cancer progression by activating epithelial–mesenchymal transition (EMT), cell cycle, and angiogenesis by sponging miRNAs (13).

HLA complex group 11 (HCG11) is a recently identified lncRNA closely associated with gastric tumors (14), lung cancer (15), glioma (16), and cervical cancer (17); however, its roles of HCG11 in various tumors are distinct. Moreover, the correlation between HCG11 and NPC is undefined, and the underlying mechanism has not yet been studied. Understanding the expression pattern and regulatory mechanisms of HCG11 may contribute to new prognostic markers and potential therapeutic targets for NPC.

miRNAs are highly conserved single-stranded noncoding RNAs of 20–23 nucleotides in length (18), which have been found to modulate the progression of various tumors, such as proliferation, apoptosis, and invasion, by downregulating oncogenes or tumor suppressor genes (19). miRNAs can partially bind mRNA at the 3-UTR (complementary sequences), which results in the blocking and degradation of translation of the target genes (18, 19). For example, microRNA-139 can negatively regulate KPNA2 to suppress hepatoma cell progression (20). MiR-9 is involved in promoting EMT and invasion by regulating LZTFL1 and PTEN. MiR-20a and miR-93 can induce angiogenesis in breast cancer by targeting VEGF. miR-21 suppresses breast tumorigenesis and angiogenesis by targeting the VEGF/VEGFR2/HIF1α axis (21). Ectopic expression of miR-490-3p is found in lung adenocarcinoma (22), esophageal cancer (23), colorectal carcinoma (24) and other tumors, which is associated with the clinical outcomes (25).

However, the miR-490-3p level in NPC is unknown, and the interaction between HCG11 and miR-490-3p, and also the underlying mechanism, are also largely undefined.

This study aimed to determine HCG11 levels in NPC and elucidate its role in NPC. The results showed that HCG11 was aberrantly expressed in NPC tissues compared to matched adjacent normal tissues. Moreover, HCG11 expression positively correlated with tumor stage and lymphatic metastasis. In contrast, NPC patients with higher HCG11 levels had shorter survival times. A mechanistic study showed that HCG11 promotes NPC progression by regulating mitogen-activated protein kinase MAPK kinase kinase 9 (MAP3K9) signaling by competitively sponging miR-490-3p. Our study reveals that HCG11 participates in the progression of NPC and may be a promising therapeutic target and a new diagnostic marker for NPC.

## Material and Methods

### Clinical Specimens

NPC tissues from 126 patients with NPC underwent primary surgery without any preoperative local or systemic antitumor treatment. All the enrolled cases were histopathologically confirmed. Studies on human NPC specimens were approved by the Ethical Committee of the Affiliated Hospital of Zhengzhou University (Henan, China).

### Cell Culture

Human NPC cell lines (5-8F, CNE-1, CNE-2) and nasopharyngeal epithelial cell line (NP69) were purchased from the National Collection of Authenticated Cell Cultures and authenticated by STR profiling. Cells were contained in RPMI1640 medium (C11875500BT, Gibco) with 10% FBS (C0232, Gibco) and 1% PS (V900929, Sigma-Aldrich). Cells were cultured at 37°C, 5% CO_2_ incubator.

### Bioinformatics Analyses

The interaction between lncRNAs and miRNAs was obtained from the Starbase database. The KEGG pathway enrichment analysis was performed for target mRNAs downstream of miR-490-3p by the “Clusterprofiler” package in R version 4.0.2. The target genes of miR-490-3p were acquired using TargetScan, miRDB, and miRTarBase.

### Q-PCR

Total RNA from cell lines and fresh NPC tissues (n = 12) was extracted using TRIzol reagent (15596026, Invitrogen). A QuantiTect Reverse Transcription Kit (205311, QIAGEN) was used for cDNA synthesis from the extracted RNA. SYBR Green Real-time PCR Master Mix (QPK-201, Solarbio) was used for qPCR. *HCG11*, *miR-490-3p*, *miR-1297*, *miR-455-5p*, *MAP3K9*, *CLCC1*, *NUFIP2*, *PAPPA*, *RBPJ*, and *SMARCD4* levels were normalized (**Table S1**).

### Cell Transfection

NC siRNA, HCG11 siRNA, NC shRNA, HCG11 shRNA, miRNA mimics, and inhibitor were obtained from the RiboBio Co. Ltd. HCG11 siRNA sequence: GAATATCTGAGGTGACAAT. NPC cells were transferred using Lipofectamine (13778030, Thermo Fisher) for the indicated times. In addition, tumor cells were transduced with control pLKO.1 lentivirus or lentivirus encoding HCG11 shRNA, and stably expressing cells were screened using puromycin.

### Fluorescence *In Situ* Hybridization (FISH)

An RNA FISH kit (430601, Gene Pharma) was used for lncRNA expression analysis in NPC according to the instructions of the manufacturer. Briefly, tissue sections were dewaxed, treated with protease, preheated, and hybridized with HCG11 FISH probes (RiboBio) overnight. Thereafter, the tissue sections were washed and stained with DAPI. The images were captured using a fluorescence microscope. The HCG11 score was determined by multiplying the intensity of immunostaining and the percentage of HCG11-positive cells.

### Colony Formation Assay

NPC cells were seeded in culture plates (500/well) for 1 week. Thereafter, tumor cells were washed for three times, fixed with paraformaldehyde (PFA, P0099, Beyotime) for 10 min, and stained for 10 min with 0.1% crystal violet (548-62-9, ChenSrc). Images were acquired and quantified by analyzing the colon numbers and size.

### CCK8 Assay

For the CCK8 assay, NPC cells were inoculated in 96-well culture plates (1000/well) and cultured for four days. The proliferation rate of NPC cells was determined by the CCK8 (CK04, Dojindo). CCK8 cells were incubated for 1 h and tested every 24 h using a microplate reader.

### Cell Apoptosis Analysis

NPC cells were transfected with NC siRNA orHCG11 siRNA for 48 h. Thereafter, cells were incubated with or without cisplatin (HY-17394, MCE, 30 μM). Cell apoptosis was determined using an Annexin V-FITC/PI Kit (A211-01, Vazyme) according to the instructions of the manufacturer.

### Transwell Assay

Briefly, cells were starved for 12 h in medium without serum, and 3 × 10^3^ cells were digested and then added to the upper compartment of Transwell (3422, Corning) with 200 μl medium containing 1% FBS. The lower compartment was filled with medium (with 10% FBS). After culturing for 48 h, NPC cells were removed from the inner membrane. The upper Transwell insert was fixed with PFA for 10 min, stained for 10 min with 0.1% crystal violet. The cells on the outer membrane were imaged under a microscope and analyzed.

### Tumor Xenograft Models

First, 5-8F cells stably overexpressing the HCG11 shRNA-pLKO.1 vector were generated. For the *in vivo* assay, 1 × 10^6^ cells (WT, HCG11 shRNA) were subcutaneously injected into female BALB/c nude mice (7 weeks-old). The tumor volume was measured daily. Mice were euthanized 19 days after injection. Thereafter, tumors were obtained for further analysis. Animal experiments were approved by the Committee of Use of Animal Care at the Affiliated Hospital of Zhengzhou University.

### H&E and Immunohistochemistry

Tumor tissue slices were prepared to prepare paraffin sections. The slices were subsequently subjected to H&E staining and immunostaining for Ki67 (9129, Cell Signaling Technology) by Servicebio Co., Ltd.

### Dual‐Luciferase Reporter Assay

The binding sites of three miRNAs (miR-490-3p, miR-1297, and miR-455-5p) in HCG11 were cloned into the PGL3-CMV-LUC-plasmid (Promega) and constructed as PGL3-CMV-LUC-lncRNA HCG11 WT (HCG11-WT) or PGL3-CMV-LUC-lncRNA HCG11 mutant (HCG-Mut) plasmids. The MAP3K9 promoter containing miR-490-3p binding sites was cloned into the PGL3-CMV-LUC-plasmid (Promega) and constructed as PGL3-CMV-LUC-MAP3K9. Thereafter, 5-8F cells were transfected with PGL3-CMV-LUC and pRLTK (Promega) with miRNA mimics, miRNA inhibitors, or miR-NC using Lipofectamine 2000. A dual-luciferase reporter assay kit (DL101-01, Vazyme) was used to detect the luciferase activity.

### Western Blot

Total protein was extracted from RIPA-lysed cells. Proteins were subjected to SDS-PAGE (P0012A; Beyotime), conducted transmembrane with polyvinylidene fluoride (IPVH00010; Millipore), blocked with milk, and incubated with primary antibodies at 4°C overnight. The primary antibodies used were anti-MAP3K9 (ab154506, Abcam) and anti-alpha tubulin (ab18251, Abcam). After primary antibody incubation, secondary antibody incubation was conducted for 1 h. The protein bands were analyzed using a chemiluminescence system (GE Healthcare).

### Statistical Analyses

All data are presented as mean ± SEM. The Student’s t-test was used to analyze the differences between the two groups. One-way analysis of variance was used to analyze the differences among multiple groups. p <0.05 was considered statistically significant.

## Results

### HCG11 Is Highly Expressed in the NPC Tissues and NPC Cell Lines

**Figure 1:**
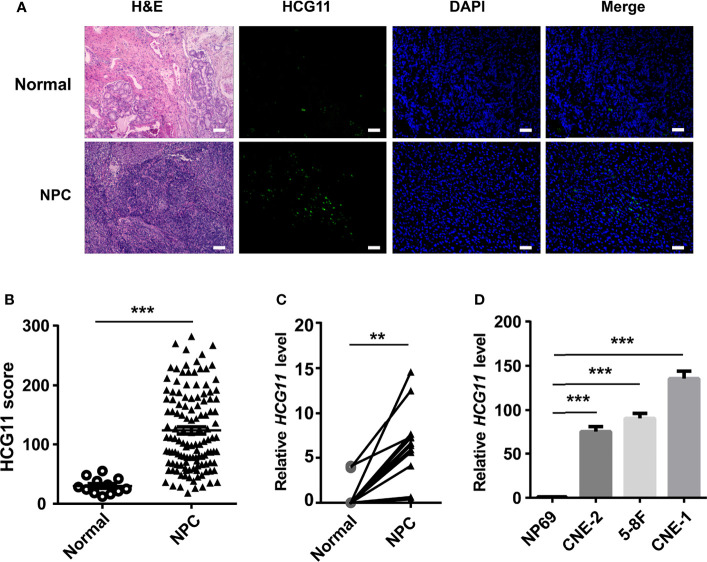
HCG11 is highly expressed in the NPC tissues and NPC cell lines. **(A, B)** FISH analysis of HCG11 level in the NPC pathological tissues (n = 126) and normal adjacent tissues (n = 12).Scale bar, 20 μm. **(C)** QPCR analysis of HCG11 in fresh NPC tissue samples and adjacent tissues (n = 12 per group). **(D)** QPCR analysis of HCG11 expression in epithelial cells NP69 and NPC cell lines (5-8F, CNE-1, CNE-2). Data were presented as Mean ± SEM. **p < 0.01, ***p < 0.001. These experiments were repeated twice **(D)**.

First, this study used FISH to test HCG11levels in NPC pathological tissues (n = 126) and normal adjacent tissues (n = 12). FISH analysis revealed that HCG11 was highly expressed in NPC tissues (**Figures 1A, B**), which was further verified by qPCR in fresh NPC samples (n = 12) and normal adjacent tissues (n = 12) (**Figure 1C**). HCG11 levels were consistently elevated in NPC cell lines (CNE-1, CNE-2, and 5-8F) compared to those in normal NP69 cells (**Figure 1D**).

### High-Expressed HCG11 Is Associated With Poor Prognosis in NPC

**Figure 2:**
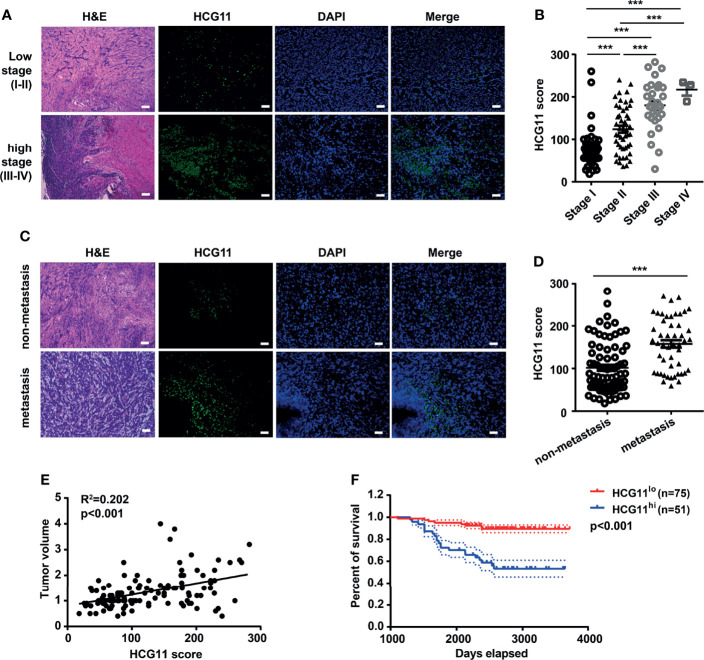
High-expressed HCG11 is associated with poor prognosis in NPC. **(A–D)** H&E staining, FISH, and quantitative analysis of HCG11 expression in the NPC tissues with low stage (stage I, n = 45; stage II, n = 48), high stage (stage III, n = 30; stage IV, n = 3) **(A, B)** and also non-metastasis (n = 77) and metastasis (n = 49) **(C, D)**.Scale bar, 20 μm. **(E)** Correlation between HCG11 expression and tumor volume was performed in NPC tissues. **(F)** Survival curve of NPC patients with different HCG11 levels (low HCG11, n = 75; high HCG11, n = 51) was shown. Data were presented as Mean ± SEM. ***p < 0.001.

To investigate the potential clinical significance of HCG11, we measured the level of HCG11 in clinical NPC tissues (n = 126) and analyzed the correlations between HCG11 and clinicopathological parameters of NPC patients (**Table 1** and **Figure 2**). We found that HCG11 was dramatically higher in advanced-stage NPC tumors (**Figures 2A, B**) and lymphatic metastasis (**Figures 2C, D**). Correlation analysis showed that HCG11 expression was positively associated with tumor size (**Figure 2E**). NPC patients with higher HCG11 levels showed a poor prognosis and shorter survival time (**Figure 2F**). The results indicate that HCG11 may be an important prognostic marker for patients with NPC.

**Table 1:** High and low HCG11 expression in nasopharyngeal carcinoma in the study population (n = 126) and stratified according to demographic and clinical variables.

Pathological feature	LncRNA HCG11			p-value
	Sample amount	Low expression	high expression	
Sex				
Male	81	52	29	p >0.05
Female	45	23	22
Age (y)				
<55	75	41	34	p >0.05
≥55	51	34	17
Tumor size				
≤1.5 cm	80	61	19	p <0.01
>1.5 cm	46	14	32
AJCC stage				
I–II	93	71	22	p <0.001
III–IV	33	4	29
Lymph node metastasis				
N0	87	58	29	p <0.001
N1–N2	39	17	22
Distant metastasis				
M0	100	61	39	p <0.001
M1	26	14	12

AJCC, American Joint Committee on Cancer.

### HCG11 Silencing Suppresses Proliferation and Migration in NPC

**Figure 3:**
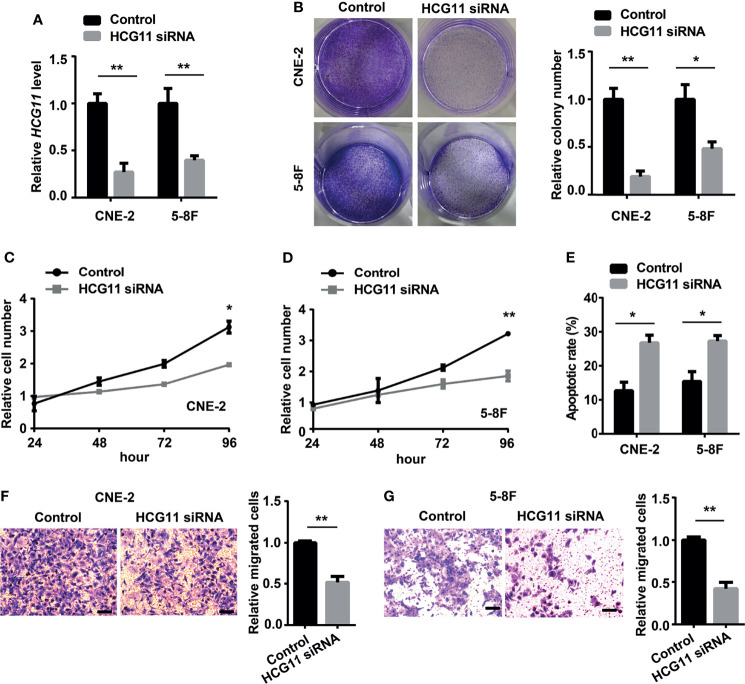
HCG11 silencing suppresses proliferation and migration in NPC cells. **(A)** QPCR analysis of HCG11 expression in 5-8F and CNE-2 cells transferred with control or HCG11 siRNA. **(B–D)** Proliferation capacity of 5-8F and CNE-2 cell lines transferred with control or HCG11 siRNA was confirmed with colony formation **(B)** and CCK8 assay **(C, D)**. **(E)** Flow cytometry analysis of the apoptosis of 5-8F and CNE-2 cells transferred with control or HCG11 siRNA. **(F, G)** Transwell assay examined cell migration of 5-8F **(F)** and CNE-2 cells **(G)** transferred with control or HCG11 siRNA. Scale bar, 50 μm. Data were presented as Mean ± SEM. *p < 0.05, **p < 0.01. These experiments were repeated three times.

The biological functions of HCG11 in NPC were further explored. HCG11 siRNA was transfected into 5-8F and CNE-2 cells and found that HCG11 expression was significantly silenced (**Figure 3A**). Colony formation and CCK8 assays showed that cell proliferation capacity was inhibited by silencing HCG11 in 5-8F and CNE-2 cells (**Figures 3B–D**). Moreover, flow cytometry analysis indicated that HCG11 knockdown significantly increased the apoptotic rate of 5-8F and CNE-2 cells (**Figure 3E**). We found a positive correlation between HCG11 expression and lymph node metastasis (**Figures 2C, D**) and further investigated the role of HCG11 in cell migration. Transwell assays showed that 5-8F and CNE-2 cells with HCG11 silencing exhibited lower migration ability (**Figures 3F, G**).

**Figure 4:**
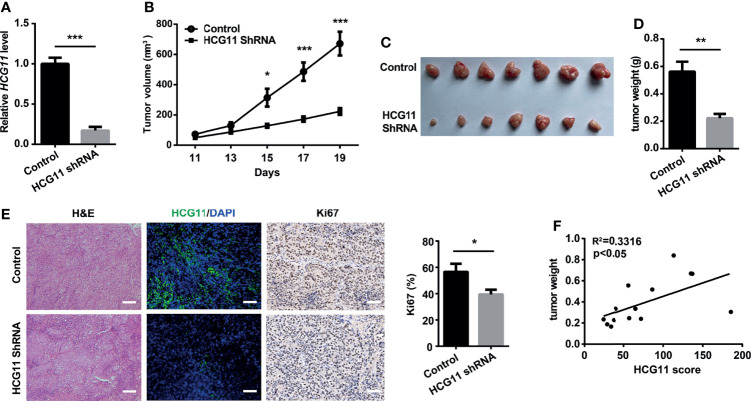
HCG11 knockdown inhibited NPC tumor progression in nude mice. Control or HCG11shRNA-transfected 5-8F cells **(A)** were inoculated into the backs of nude mice. **(B–D)**Tumor growth curve **(B)**, tumor graph **(C)**, and tumor weight **(D)** in HCG11-shRNA and control 5-8F tumors are shown. **(E)** H&E, HCG11, and Ki67 staining are shown in 5-8F tumors. Scale bar, 50 μm. **(F)** Correlation analysis of HCG11 levels and tumor weight in 5-8F tumors. Data are presented as mean ± SEM. *p < 0.05, **p < 0.01, ***p < 0.001. n = 7 mice per group.

We also investigated the role of HCG11 in NPC progression *in vivo*. First, we stably transfected 5-8F cells with the pLKO.1-empty vector and pLKO.1-HCG11-shRNA vector (**Figure 4A**). Control 5-8F or HCG11-shRNA 5-8F cells (1 × 10^6^ cells) were inoculated subcutaneously into BALB/c nude mice. During the establishment of the 19 days, 5-8F cells with HCG11 knockdown showed a decreased growth rate (**Figure 4B**), which was further confirmed by the smaller average volume and lighter tumor weight in the HCG11shRNA group (**Figures 4C, D**). HCG11 expression in resected tumor tissues was significantly downregulated in the HCG11 shRNA group, as detected by FISH. The percentage of Ki67-postive cells was reduced in HCG11 knockdown tumor tissues, as determined by immunohistochemistry (**Figure 4E**). Moreover, we found a positive correlation between HCG11 levels and tumor weight (**Figure 4F**), which is consistent with previous results (**Figure 2E**). In summary, these results indicated that HCG11 plays an oncogenic role in NPC.

### miR-490-3p Is a Downstream Target of HCG11

**Figure 5:**
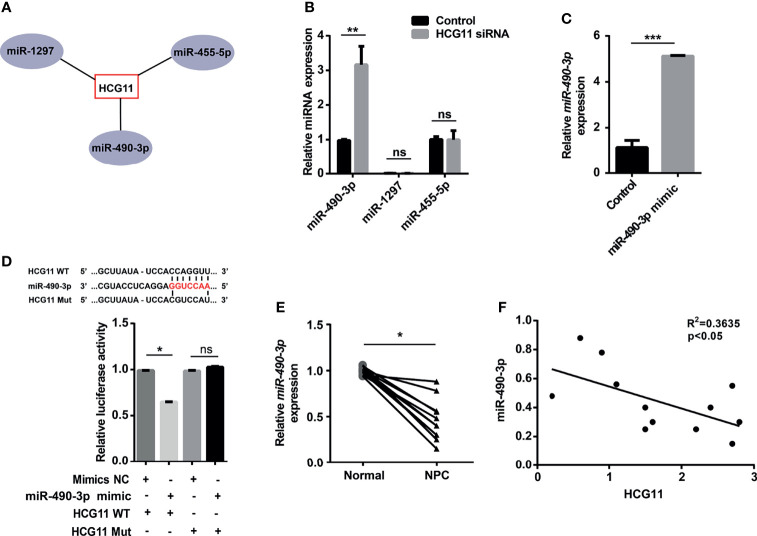
miR-490-3p is a downstream target of HCG11. **(A)** Bioinformatics analysis of miRNAs targets of HCG11. **(B)** QPCR analysis of miR-490-3p, miR-1297 and miR-455-5p level in 5-8F cells transferred with HCG11 siRNA and control siRNA. **(C)** QPCR analysis of miR-490-3p in 5-8F cells (Control, miR-490-3p mimic). **(D)** 5-8F cells were co-transfected with PGL3-CMV-LUC-lncRNA HCG11 WT (HCG11 WT) or PGL3-CMV-LUC-lncRNA HCG11 mutant (HCG Mut) and control or miR-490-3p mimic. Luciferase activity of HCG11 WT or HCG11 Mut was determined. **(E)** QPCR analysis of miR-490-3p in NPC tissues and adjacent tissues (n=12). **(F)** Correlation between HCG11 expression and miR-490-3p was performed in NPC tissues. Data were presented as Mean ± SEM. Ns, p > 0.05, * p < 0.05, ** p < 0.01, *** p < 0.001. These experiments were repeated for three times **(B–D)**.

It has been established that lncRNAs can sponge miRNAs to regulate specific genes and affect cancer malignant phenotypes (7), whereas the correlation between miRNAs and HCG11 in NPC remains to be explored. In this section, we first obtained the interaction between HCG11 and miRNA from the StarBase database and database analysis. The results showed that miR-490-3p, miR-1297, and miR-455-5p are potential targets of HCG11 (**Figure 5A**). qPCR analysis revealed higher levels of miR-490-3p, but not miR-455-5p or miR-1297, were observed in 5-8F and CNE-2 cells transfected with HCG11 siRNA (**Figure 5B**). To further confirm whether miR-490-3p is a target of HCG11, we co-transfected miR-490-3p mimics with PGL3-CMV-LUC.miR-490-3p expression was significantly upregulated, and the luciferase activity of HCG11 WT was significantly inhibited, but not the HCG11 mutant (**Figures 5C, D**). The luciferase reporter assay further suggested that HCG11 does not contain binding sites for miR-455-5p or miR-1297 (**Figure S1**). Moreover, we confirmed the correlation between HCG11 and miR-490-3p in NPC tissues and found that miR-490-3p was downregulated in NPC tumors (**Figure 5E**), which was negatively associated with HCG11 (**Figure 5F**). In summary, these results indicated that miR-490-3p is negatively regulated by HCG11.

### Oncogenic Function of HCG11 Was Partially Reversed by miR-490-3p in NPC Cells

**Figure 6:**
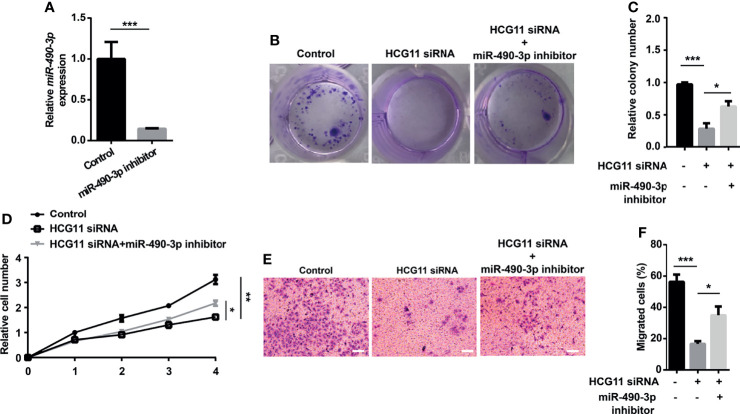
Oncogenic function of HCG11 was partially reversed by miR-490-3p in NPC cells. **(A)** QPCR analysis of miR-490-3p in 5-8F cells (control, miR-490-3p inhibitor). **(B–D)** Proliferation capacity of 5-8F cells transferred with miR-490-3p inhibitor or HCG11 siRNA was confirmed using colony formation assay **(B, C)** and CCK8 assay **(D)**. **(E, F)** Cell migration of 5-8F cells transferred with miR-490-3p inhibitor or HCG11 siRNA was confirmed by transwell assay. Data were presented as Mean ± SEM. * p < 0.05, ** p < 0.01, *** p < 0.001. These experiments were repeated for three times.

We then investigated whether HCG11 regulated NPC progression by inhibiting miR-490-3p. In colony formation and CCK8 assays, cell proliferation capacity was significantly inhibited by downregulating HCG11 in 5-8F cells, which could be partially rescued by miR-490-3p inhibitor (**Figures 6A–D**). We also investigated the role of HCG11/miR-490-3p in cell migration. 5-8F cell lines transfected with HCG11 siRNA showed lower migration ability compared to the control siRNA groups, while the miR-490-3p inhibitor significantly reversed the impaired migration ability of 5-8F cells with HCG11 silencing (**Figures 6E, F**). Therefore, HCG11 can regulate NPC cell proliferation and migration *via* the negative regulation of miR-490-3p.

### HCG11 Regulates NPC Cells *via* miR-490-3p/MAP3K9 Signaling

**Figure 7:**
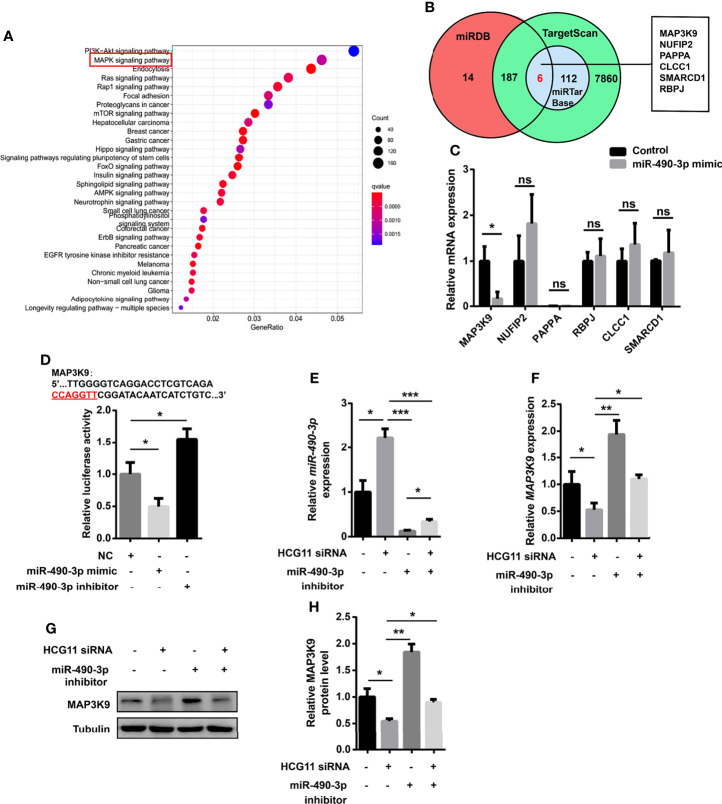
HCG11 regulates NPC cells via miR-490-3p/MAP3K9 signaling. **(A)** KEGG pathway enrichment analysis of target mRNAs downstream of miR-490-3p were shown. **(B)** Venn diagram analysis of miR-490-3p related genes was listed. **(C)** Q-PCR analysis of gene expression in 5-8F cells transferred with control or miR-490-3p mimic. **(D)** 5-8F cells were co-transfected with PGL3-CMV-LUC-MAP3K9 and miR-490-3p mimic or miR-490-3p inhibitor. Luciferase activity of MAP3K9 was determined. **(E, F)** Q-PCR analysis of miR-490-3p **(E)** and MAP3K9 **(F)** in 5-8F cells transferred with miR-490-3p inhibitor or HCG11 siRNA. **(G, H)** The protein expression of MAP3K9 was confirmed in 5-8F cells transferred with miR-490-3p inhibitor or HCG11 siRNA. Data were presented as Mean ± SEM. Ns, p > 0.05, * p < 0.05, ** p < 0.01, *** p < 0.001. These experiments were repeated for three times **(C–H)**.

It is known that ncRNA–mRNA interactions are involved in various tumor processes (7). miRNAs bind to the 3-UTR of mRNAs at partially complementary sequences to regulate the blocking/degradation of mRNA translation. To confirm the oncogenic genes, the KEGG pathway enrichment analysis was performed for target mRNAs downstream of miR-490-3p by the “Clusterprofiler” package in RStudio. We also identified the downstream targets of miR-490-3p using TargetScan, miRDB, and miRTarBase. A bioinformatics analysis showed that MAP3K9 may be a potential target of miR-490-3p (**Figures 7A, B**). As a serine/threonine protein kinase, MAP3K9 functions as an upstream activator of MAPK signaling, which is involved in tumor progression. A qPCR analysis revealed that the miR-490-3p mimic significantly downregulated MAP3K9 (**Figure 7C**). To confirm the relationship of miR-490-3p and MAP3K9, we co-transfected miR-490-3p mimic or miR-490-3p inhibitor with PGL3-CMV-LUC-MAP3K9, the luciferase activity of MAP3K9 was significantly inhibited by miR-490-3p mimic and enhanced by amiR-490-3p inhibitor (**Figure 7D**).

To further investigate whether HCG11 regulates miR-490-3p/MAP3K9 signaling in NPC cells, we co-transferred HCG11 siRNA and miR-490-3p inhibitor in 5-8F cells. MiR-490-3p was significantly inhibited by the miR-490-3p inhibitor (**Figure 7E**) and thus upregulated MAP3K9 levels (**Figures 7F–H**). HCG11 knockdown upregulated miR-490-3p expression (**Figure 7E**) and significantly downregulated MAP3K9 expression (**Figures 7F–H**). In summary, HCG11 regulates NPC cells through miR-490-3p/MAP3K9 signaling.

## Discussion

Studies focusing on lncRNAs regulating and integrating oncogenes have been widely conducted, providing significant implications for understanding tumor progression. Approximately 60,000 lncRNAs have been identified, which account for more than 50% of the cellular transcriptome; however, only less than 10% have been demonstrated aberrantly expressed and functional in cancers (9). However, the effect of HCG11 on cancer remains controversial. This study found that lncRNA HCG11 was highly expressed in NPC and promoted tumor progression by regulating MAP3K9 signaling by competitively sponging miR-490-3p.

Through FISH analysis of NPC and normal tissues, we found that HCG11 was significantly overexpressed in NPC tumors, which was identified by qPCR analysis of HCG11 levels in fresh NPC samples and cells. We further divided patients according to TNM and found that HCG11 was dramatically higher in NPC tumors with advanced stage and lymphatic metastasis but was negatively associated with the survival of NPC patients. *In vitro* and *in vivo* experiments showed that HCG11 knockdown significantly reduced cell proliferation, which was consistent with the high apoptosis rate and decreased migratory ability of NPC cells, demonstrating the critical role of HCG11 in NPC tumorigenesis and metastasis.

Increasing evidence shows that lncRNAs regulate tumor cells through oncogenic or tumor-suppressing pathways *via* direct chromatin remodeling, post-transcriptional regulation, and translational control (26). lncRNAs can regulate cancer cells by interdicting the effects of miRNAs as miRNA sponge (9). For example, the lncRNA ARHGAP18 promotes tumor metastasis in HCC *via* miR-153-5p (11). In CRC, MCM3AP-AS1 regulates miR-193a-5p/SENP1 to promote cell activity (27). HNF1A-AS1 is induced by MYC to facilitate glioma progression through miR-32-5p/SOX4 (12). The ectopic miR-490-3p expression exists in colon cancer, esophageal squamous cell carcinoma, and other cancers. The tumor inhibitory function of miR-490-3p is also found in various cancers; for example, miR-490-3p influences hepatic carcinoma by downregulating TNKS2 (28). MiR-490-3p can also inhibit biological characteristics by reducing TGFβR1 in colon cancer cells (29). Although miRNAs have been studied in various cancers, the correlation between miRNAs and lncRNAs in NPC remains unclear. qPCR and dual-luciferase reporter assays demonstrated that HCG11 could target the binding sites of miR-490-3p. MiR-490-3p silence by HCG11 reduced the proliferation and migration ability of NPC. HCG11 and miR-490-3p can compete with each other and regulate MAP3K9 signaling, providing important clues for understanding the importance of lncRNA–miRNA functional networks in cancer.

Because of their high expression and specificity in many cancers, lncRNAs can be a basis for many clinical applications in oncology (30). MIR31HG is a novel factor involved in melanoma progression (31). Increased expression of FAM83H-AS1 may predict poor prognosis in cancer patients (32). In this study, HCG11 was positively correlated with advanced stage and lymph node metastasis and negatively associated with NPC patient survival. The novel lncRNA HCG11 and mRNA biomarkers associated with clinical traits provided new insights into prognostic markers and effective treatment of NPC.

## Conclusions

HCG11 facilitates NPC progression *via* MAP3K9 signaling by sponging miRNA-490-3p, which may contribute to new prognostic markers and promising therapeutic targets.

## Data Availability Statement

The original contributions presented in the study are included in the article/**Supplementary Material**. Further inquiries can be directed to the corresponding authors.

## Ethics Statement

Studies with human nasopharyngeal carcinoma specimens have been approved by the Ethical Committee of the Affiliated Hospital of Zhengzhou University (Henan, China), and informed consents were obtained from all patients’ family. The patients/participants provided their written informed consent to participate in this study. The animal experiments were approved by the committee of Use of Animal Care in the Affiliated Hospital of Zhengzhou University. Written informed consent was obtained from the owners for the participation of their animals in this study.

## Author Contributions

JZ conceived the research. JZ and ZZ conducted the experiments. HR, YW and CZ performed data analysis. WZ and XM contributed to the revision of the manuscript. LM and XL gave the final approval. All authors contributed to the article and approved the submitted version.

## Funding

This study was supported by grants from the Henan Provincial Health and Health Commission, Medical Science and Technology, and Provincial Ministry Co-Construction Project (Grant No. SBGJ2018018). This study was also funded by the following three fundings: Natural Science Foundation of Henan (212300410395), the Science and Technology Project of Henan province of China (Grant No. 212102310620) National Natural Science Foundation of China (Grant No. 82172351).

## Conflict of Interest

The authors declare that the research was conducted in the absence of any commercial or financial relationships that could be construed as a potential conflict of interest.

## Publisher’s Note

All claims expressed in this article are solely those of the authors and do not necessarily represent those of their affiliated organizations, or those of the publisher, the editors and the reviewers. Any product that may be evaluated in this article, or claim that may be made by its manufacturer, is not guaranteed or endorsed by the publisher.
