# Supplementary material for: LncRNA HCG11 Facilitates Nasopharyngeal Carcinoma Progression Through Regulating miRNA-490-3p/MAP3K9 Axis
Source: Front Oncol. 2022 Apr 7;12:872033. doi: 10.3389/fonc.2022.872033 (PMC9021694; doi:10.3389/fonc.2022.872033)
Supplement: Supplementary file 1 [file DataSheet_1.docx]

# Supplementary data

**Figure S1. HCG11 had no binding sites of miR-455-5p or miR-1297**

**(A-B)** The relative luciferase activity was detected in 5-8F cells co-transfected with PGL3-CMV-LUC-lncRNA HCG11 WT (HCG11 WT) and miR-1297 mimics (A) or miR- miR455-5p mimics (B). Data were presented as Mean ± SEM. Ns, p＞0.05. Data were repeated for three times.

**Table S1. Primers**

| Gene | Primers |
| --- | --- |
| *HCG11* (F) | GGTGACCACTCTGTCGCCATTG |
| H*CG11* (R) | GATCGCCGCTAACCGTTCCAC |
| *miR-490-3p* (F) | CGCAACCTGGAGGACTCC |
| *miR-490-3p* (R) | AGTGCAGGGTCCGAGGTATT |
| *miR-455-5p* (F) | CGCGTATGTGCCTTTGGACT |
| *miR-455-5p* (R) | AGTGCAGGGTCCGAGGTATT |
| *miR-1297* (F) | CGCGCGCGTTCAAGTAATT |
| *miR-1297* (R) | AGTGCAGGGTCCGAGGTATT |
| *MAP3K9* (F) | GGAGGTGGTTGAGCATGACTAATGG |
| *MAP3K9* (R) | TGGCAGACAGCATCAGACAAGTTAAG |
| *CLCC1* (F) | CCACATTTGTAACGGAGCCATTGAAG |
| *CLCC1* (R) | CCAGCACCATAGCAGAAACTCAGG |
| *NUFIP2* (F) | ATTCTATTGCCCAACTGGTGCTCTC |
| *NUFIP2* (R) | AGGACAGCCACTAACAGACTAAGAAC |
| *PAPPA* (F) | GACCACAACAGCGAGTCCATCATC |
| *PAPPA* (R) | TGAGGATACCACTTGAGACCAGCAG |
| *RBPJ* (F) | GCATTCCGAGAAGGTTGGAGATGG |
| *RBPJ* (R) | GGTTCTGGTGTGTAGGTAAAGGTAAGG |
| *SMARCD4* (F) | AGCAGAGACGACAAGAATTAGAGCAAG |
| *SMARCD4* (R) | GCAAGACCAAGGCAGATACTATGAGG |
| *GAPDH* (F) | CAGGAGGCATTGCTGATGAT |
| *GAPDH* (R) | GAAGGCTGGGGCTCATTT |
| *U6* (F) | CGCTTCGGCAGCACATATAC |
| *U6* (R) | CGAATTTGCGTGTCATCCTT |
