# Supplementary figures and images for: LncRNA HCG11 Facilitates Nasopharyngeal Carcinoma Progression Through Regulating miRNA-490-3p/MAP3K9 Axis
Source: Front Oncol. 2022 Apr 7;12:872033. doi: 10.3389/fonc.2022.872033 (PMC9021694; doi:10.3389/fonc.2022.872033)

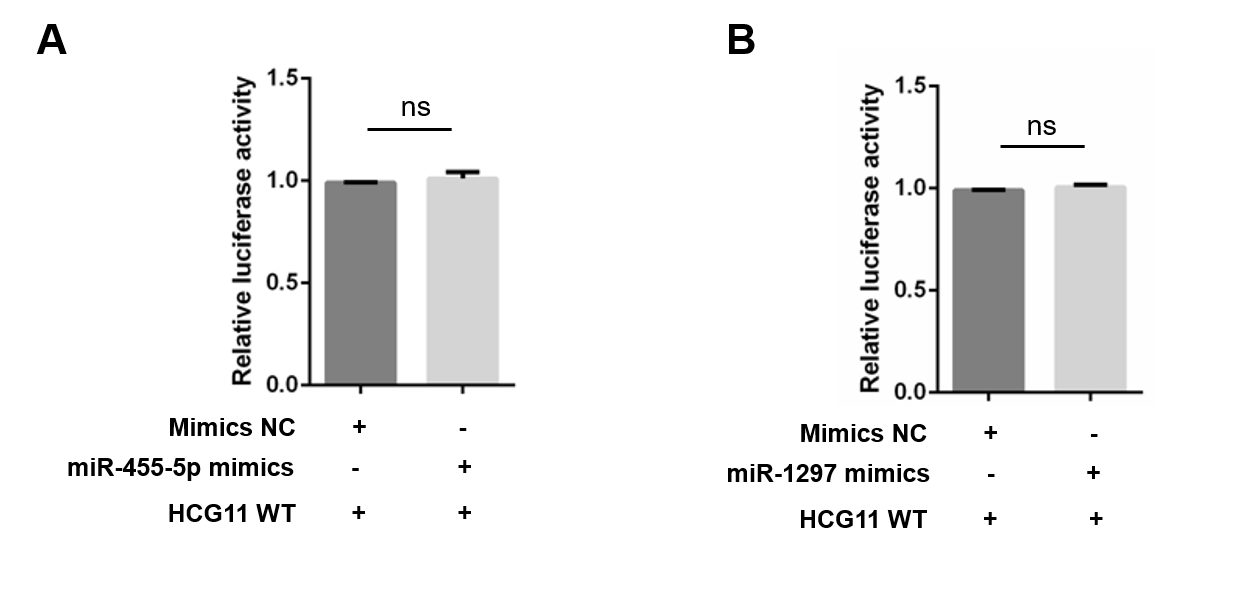

Supplement: Supplementary file 2 [file Image_1.tif]
